# Some Effects of Tight Lacing on Certain of the Abdominal Contents

**Published:** 1895-12-07

**Authors:** J. C. Webster

**Affiliations:** Assistant to Professor of Midwifery and Diseases of Women, University of Edinburgh.


### Medical Progress and Hospital Clinics.

[*The Editor will be glad to receive offers of co-operation and contributions from members of the profession. All letters should be addressed to* The Editor, at the Office, 428, Strand, London, W.C.]

### SOME EFFECTS OF TIGHT LACING ON CERTAIN OF THE ABDOMINAL CONTENTS.

By J. C. Webster, M.D., F.R.C.P.E., Assistant to Professor of Midwifery and Diseases of Women, University of Edinburgh.

For a considerable number of years there has been much extravagant writing on this subject. Ever a favourite topic with the popular hygienist, it has reached a foremost place in the stock-in-trade of the platform teacher and of the writers of medical tracts. The accumulated efforts of many fertile imaginations have created a well-defined list of pathological changes and clinical phenomena resultant upon this much-abused dress habit, and there can be no doubt that the published accounts of these horrors have proved an active stimulus to those engaged in fighting the cause of rational clothing for the female sex.

Of elaborate and dispassionate scientific inquiries there are few accounts, and these have not been at all widely noticed by medical writers. Until recently the chief investigations have been those of Sœmmerring, Cruveilhier, Frerichs, and Engel.

I desire in this paper to draw attention to some of the conditions found *post mortem*. These have lately been fully described by Dr. Paul Hertz, of Copenhagen, in a paper published in Germany in 1894. He made a most careful study of fifty cadavera, and his descriptions form a valuable work of reference. Its perusal makes it evident that the pathology of tight-lacing is by no means a definite and simple matter. Many variations are found in the degree to which the abdominal viscera are affected. The explanation of the mechanism by which some of the changes are brought about is not always clear. Several points yet require investigation; and in particular, it may be stated that careful inquiries should be made for the purpose of determining the results of tight-lacing in women under different conditions—viz., during the growing period, in the adult state, in relation to child-bearing, and in relation to occupation. At present we can only give some of the general results obtained. It will be best to mention these in connection with the viscera separately.

**The Liver.**—The changes met with in this organ are of a twofold nature—viz., as regards shape and position. Two main types of deformity are found.

In one the liver reaches further down than normally; its antero-posterior diameter is diminished; its anterior part is somewhat flattened and thinned, hanging downwards like a flap for several inches. On the anterior surface is a transverse or oblique furrow, whose margins are thickened by inflammation in the liver-capsule. The shape of the organ, as seen from the front, varies somewhat; it may be irregularly triangular or quadrilateral. When the liver extends far down a very deep notch usually forms between the right and left lobes. This is due to the resistance of the ligamentum teres, or obliterated umbilical vein, against the soft liver-substance. The posterior surface is considerably altered from the normal. On the right side it may be deeply grooved by the anterior surface of the right kidney. This organ, of course, in normal cases, only touches the under surface of the liver. At the upper and lower ends of this groove the liver-substance bulges backwards over the ends of the kidney. Hertz describes the kidney in this position as being in a nest in the liver. When the colon is displaced downwards considerably the projection of the liver below the kidney becomes wedged between the bowel and the latter organ. In this condition of matters the kidney may be very little displaced downwards, or not at all.

In the other type a different condition exists. The liver is wider and thicker, especially in its upper part. It is curved so that its concavity looks towards the spine. In marked cases the left lobe may extend around so as somewhat to embrace the stomach, and even the spleen. The anterior surface is moulded by the body-wall. It is curved from side to side, and when the lower part of the liver is much thinner than the upper part the anterior surface resembles somewhat the surface of a cone. The lower margin is usually more transverse than in normal cases. A short distance above this a small furrow is found, generally accompanied with perihepatitis. Below this the liver-substance is usually considerably atrophied, especially near the gall-bladder. In some cases it may be reduced to fibrous tissue.

The posterior surface of the liver has the following relationships. The extreme right portion lies largely in the lumbar region, its lower part in contact with the right kidney, which is pushed downwards, its lower end being tilted forwards. The central portion is displaced downwards, so that the lobus spigelii touches colon, pancreas, or small intestine, most commonly the duodenum. The left lobe covers, as a rule, the body of the pancreas, and also the cardiac end of the stomach; often it extends around to cover the spleen.

Several variations are found from these pure types, *e.g.*, the extension downwards of a three-cornered flap-like projection of the anterior wall. Mixed forms are also found.

**The Gall Bladder.**—Various changes in the gall bladder are found. Very frequently it is dilated, the wall being often thickened. It may be somewhat pressed upon by the right kidney when the latter is displaced, so that its fundus is pushed forwards. In some cases it may be in contact with the duodenum and head of the pancreas; in others with the pylorus.

When the bladder is much lengthened its fundus may, in certain cases, bend over the edge of the liver, or it may turn down over the displaced lower end of the kidney or over the duodenum. The cystic duct is very much curved in some cases.

**The Spleen.**—Slight dislocations of the spleen are found in some cases; marked ones are rare. Hertz describes in particular a rotation of the organ, whereby the under surface, which is in contact with the left kidney is turned forwards. This change is probably brought about by the pressure of the stomach or of the left lobe of the liver.

**The Pancreas.** — Normally, this organ is rather securely fixed in position. It is surrounded by viscera which are more or less moveable, and it is found that dislocations in these affect the pancreas. Hertz draws particular attention to the downward displacement of the body, that part of the organ lying between the head and the spleen, which has a more or less horizontal position.

When downward dislocation of the left lobe of the liver and stomach occurs, along with descent of the transverse colon, a rotation of the pancreas is brought about whereby the upper surface is made to look forwards and the anterior surface downwards. This is most marked in its outer part. When much pressure on the organ is caused by these viscera, it becomes flattened.

Another displacement occurs to the head of the pancreas. Normally, this lies to the right of the spine, covering the inferior vena cava.

In abnormal cases, owing to the pressure of an enlarged right lobe of the liver somewhat pushed downwards, or to that of an enlarged gall-bladder, the head may be displaced towards the left. When the lower end of the left kidney also is dislocated forwards, it may cause pressure against the head of the pancreas. Marked downward descent of the pancreas as a whole is not often found. In most cases it is lower than normal, but the variations are considerable. Hertz has found it pushed downwards the thickness of a vertebral body.

The relations of the organ to surrounding structures are considerably altered. When the left lobe of the liver is elongated and displaced downwards, more of it covers the pancreas than normally. When the pylorus and first part of the duodenum are pushed downwards, more of the head of the pancreas is brought into relation with the quadrate lobe of the liver. When the transverse colon is lower, the lower part of the head may be easily seen through the thinned meso-colon. In this case also the upper part of the anterior surface is separated from the anterior abdominal wall, only by the left lobe of the liver, if it be elongated, and by the lesser omentum.

Very often adhesions are found between the pancreas and the posterior wall of the stomach.

**The Stomach.**—The most constant change is a downward displacement. Abnormalities in the position, shape, and size of the left lobe of the liver are the most important factors in bringing about altered relationships in the stomach.

The most frequent change is a displacement downwards and to the left, whereby a sharp angle is formed in the lesser curvature; the upper part of the stomach is then directed from the cardiac orifice downwards and outwards, the lower part extends transversely or obliquely across the left epigastrium, the pyloric orifice being directed towards the right. Many variations are found in the extent to which the stomach is altered. In cases in which the left lobe of the liver is much enlarged and displaced downwards, especially if the spleen be enlarged, the upper part of the stomach is so interfered with that besides the downward displacement of the organ there occurs a dilation of the lower portion and of the pyloric end. The organ may then have the shape of a fishing-hook.

**The Duodenum.**—Generally, the duodenum lies somewhat lower than normal, and is pushed slightly forwards. It varies according to the position which the pylorus takes as a result of displacement of the stomach. When the stomach has the shape of the fish-hook, the first part of the duodenum is stretched vertically, and there is a sharp bend at the upper end. The hepatic-duodenal ligament is often stretched. The second part of the duodenum is usually much less disturbed than the first, owing to its close union with the head of the pancreas. Both structures are usually displaced together. It is very common to find adhesions between the duodenum and gall-bladder when the latter is enlarged and displaced downwards. These adhesions may extend to the liver and to the colon. They may be very extensive.

**The Colon.**—Down-sinking of the transverse colon is common. The mesentery becomes more or less stretched. In extreme cases it may be much displaced, and may be found in front of the liver or stomach, or in other parts of the abdominal cavity. Sometimes it gets to be behind coils of small intestine. Adhesions are often formed between the colon and gall-bladder and neighbouring structures.

**The Kidneys.**—The kidneys may be more or less displaced; in this state they are generally more or less moveable, though in some cases, owing to fixation or adhesions, this characteristic may be wanting. The displacement of the right kidney in certain cases, whereby it becomes somewhat embedded in the back wall of the liver, has been described. In other cases it is simply pushed downwards more or less as a whole. In marked instances it may become very mobile, and may move in various directions behind the peritoneum. Its tendency is to move inwards rather than outwards. According to Hertz, it is rare to find a mesentery developed in connection with this mobile kidney. This kidney may be found behind the mesocolon, behind the mesentery, or in the iliac fossa. In certain cases it is impossible to return the organ to its normal position because the displaced liver lies there. Dislocations of the left kidney are much less frequent than in the case of the right. In the great majority of cases there are only slight alterations in its position. When the outer end of the pancreas is markedly displaced the kidney may be considerably dislocated.

